# Improving quality of care through patient-reported outcome measures (PROMs): expert interviews using the NHS PROMs Programme and the Swedish quality registers for knee and hip arthroplasty as examples

**DOI:** 10.1186/s12913-018-2898-z

**Published:** 2018-02-07

**Authors:** Birgit Prodinger, Paul Taylor

**Affiliations:** 1 University of Applied Sciences Rosenheim, Faculty for Applied Health and Social Sciences, Hochschulstr. 1, 83024 Rosenheim, Germany; 2 CHIME, Institute of Health Informatics, 222 Euston Road, London, NW1 2DA UK; 3 Swiss Paraplegic Research, Guido A. Zäch Str. 4, 6207 Nottwil, Switzerland

**Keywords:** Health information system evaluation, Quality registry, Oxford hip score, Oxford knee score, EQ-5D, Expert interviews, Social network analysis, Directive content analysis

## Abstract

**Background:**

Patient reported outcome measures (PROMs) have been integrated in national quality registries or specific national monitoring initiatives to inform the improvement of quality of care on a national scale. However there are many unanswered questions, such as: how these systems are set up, whether they lead to improved quality of care, which stakeholders use the information once it is available. The aim of this study was to examine supporting and hindering factors relevant to integrating patient-reported outcome measures (PROMs) in selected health information systems (HIS) tailored toward improving quality of care across the entire health system.

**Methods:**

First, a systematic search and review was conducted to outline previously identified factors relevant to the integration of PROMs in the selected HIS. A social network analysis was performed to identify networks of experts in these systems. Second, expert interviews were conducted to discuss and elaborate on the identified factors. Directive content analysis was applied using a HIS Evaluation Framework as the frame of reference. This framework is structured into four components: Organization, Human, Technology, and Net benefits.

**Results:**

The literature review revealed 37 papers for the NHS PROMs Programme and 26 papers for the SHPR and SKAR: Five networks of researchers were identified for the NHS PROMs Programme and 1 for the SHPR and SKAR. Seven experts related to the NHS PROMs Programme and 3 experts related to the SKAR and SHPR participated in the study. The main themes which revealed in relation to Organization were Governance and Capacity building; to Human: Reporting and Stakeholder Engagement; to Technology: the Selection and Collection of PROMs and Data linkage. In relation to Net benefits, system-specific considerations are presented.

**Conclusion:**

Both examples succeeded in integrating PROMs into HIS on a national scale. The lack of an established standard on what change PROMs should be achieved by an intervention limits their usefulness for monitoring quality of care. Whether the PROMs data collected within these systems can be used in routine clinical practice is considered a challenge in both countries.

**Electronic supplementary material:**

The online version of this article (10.1186/s12913-018-2898-z) contains supplementary material, which is available to authorized users.

## Background

Health care services are increasingly challenged to sustain high quality of care while facing increasing demand and financial shortcuts. Integrating the patients’ perspective in evaluating quality of health services has been urged to ensure that the patients’ judgment of health outcomes is considered in improving the quality of services [1]. Patient reported outcome measures (PROMs) are measures that provide data directly reported by the patient or the patient proxy [2] and thus reflect the patient’s perspective. PROMs can be used to evaluate the effectiveness of care, as well as safety; both aspects are considered as quality characteristics [3]. Despite the potential value of PROMs, the evidence that PROMs are included in routine clinical practice [4, 5] and quality management [6] is still scarce. While current work in health services research has focused predominantly on measures of failure such as hospital readmission or mortality rates, PROMs are promising as measures of success, such as improvement in functioning [7].

There are different approaches to collect relevant health outcomes for the monitoring of quality in health services through PROMs. One approach is to collect PROMs in routine clinical practice and use the information primarily for shared clinical decision making [8]. Clinicians consider PROMs as valuable for shared decision-making, if the purpose and the process of data collection complements routine practice and is not disruptive. Thus, if the appropriate infrastructure is not put in place, clinicians are likely to refrain from using PROMs [9–11]. If the information is aggregated at service level, it could potentially also inform the monitoring of quality of health services.

Another approach – which is of key interest for the present study – is to collect PROMs within the entire health system and use the data to monitor quality of care across hospitals. Such approach has been considered in national quality registries and specific national monitoring initiatives [1]. International societies have started to publish recommendations on how to integrate PROMs into registries [12–14]. If the information is collected already within the entire health system, PROMs data would become ideally not only available for improving quality of care but also for clinical decision making. However, how these systems succeeded in improving quality of care, whether they are suited for informing clinical practice, which stakeholders actually utilize the information once it is available within the health system, etc. remains yet to be examined. Furthermore, there may also be differences in how such systems have been set up, e.g. PROMS have been an integral part of their development or PROMs have been introduced in existing information systems later on. Therefore, there is a need to systematically examine existing systems to guide the future development of such systems, as well as the implementation in other clinical settings or countries.

The aim of this study was to examine supporting and hindering factors relevant to integrating patient-reported outcome measures (PROMs) in selected health information systems (HIS) tailored toward improving quality of care across the entire health system. To respond to this aim, the NHS PROMs Programme and the Swedish Hip Quality Register (SHPR) and Swedish Knee Arthroplasty Register (SKAR) were used as examples. These examples were chosen, since both aim to improve quality of care for the same clinical population by using PROMs and have received international recognition as evident through international peer-reviewed publications yet differ in their development. The NHS PROMs Programme is a fairly recent attempt to integrate PROMs on a national scale and is targeted toward four clinical populations. The Swedish Quality Registers often serve as reference systems given their extensive experience in running such information systems [15]. The Hip and Knee Arthroplasty Register were chosen for comparative reasons with the NHS PROMs Programme. Though the systems pursue similar goals and have the same target population, they are different in other aspects as outlined in Table 1. The NHS PROMs programme, the SHPR and SKAR are considered in this study as health information systems (HIS).

**Table 1:** Overview of selected information systems

	NHS PROMs Programme	Swedish Hip Quality Register (SHPR) and Swedish Knee Arthroplasty Register (SKAR)
Aim	Improving quality of care for four clinical populations; for the purpose of this study, the focus was on hip and knee arthroplasty.	Improving quality of care for people with hip and knee arthroplasty
Launch	2009 Launched independently from the National Joint Registry for Hip and Knee Arthroplasty which was launched in 2002	~ 2002 Integration of PROMs into Swedish Hip and Knee Arthroplasty Registers which were launched in 1979 and 1975 respectively
Data collection		
Generic PROMs:	EQ-5D and the corresponding visual analogue scale for health-related quality of life The EQ-5D is a generic instrument developed by the EuroQoL Group and contains 5 dimensions (mobility, self-care, usual activities, pain and discomfort, anxiety and depression	
Health condition specific PROMs:	Oxford Knee Score (OKS) and Oxford Hip Score (OHS)	SHPR: no health condition specific PROM SKAR: Knee injury and Osteoarthritis Outcome Score (KOOS)

## Methods

**Fig. 1:**
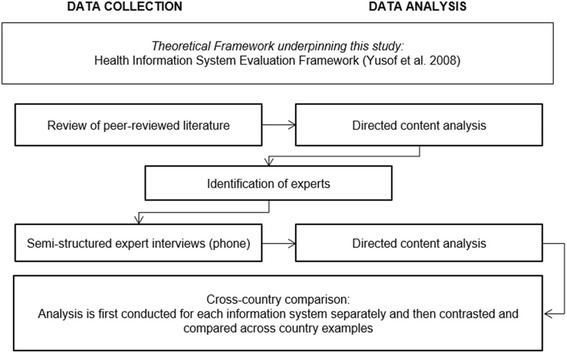
Overview of study design

First, a systematic search and review [16] of internationally peer-reviewed literature was carried out to identify supporting and hindering factors relevant to integrating PROMs as outlined previously with regards to the selected information systems. The results of this review served as the foundation for building an interview guide. Second, expert interviews were performed to discuss and elaborate on the identified factors. The study design is outlined in Fig. 1.

To account for the complexity in the evaluation of HIS [17], the Health Information System Evaluation Framework developed by Yusof et al. [18] served as the frame of reference for data analysis in this study. This framework consists of four components, each with several dimensions; the organization (incl. Structure and environment), the human (incl. System use and user satisfaction), the technology (incl. System, information and service quality), and net benefits (incl. Positive and negative impact on potential end-users) and has been recommended and used for evaluation of health information systems [19–21].

### Data collection

#### Part 1 systematic search and review

A systematic search and review is characterized by a systematic literature search and the subsequent critical review of its content to derive state of the art knowledge on a phenomenon. Such type of review incorporates any study type [16]. The literature search was conducted in December 2015, without time limit for publication dates, using the databases PubMed and SCOPUS. The search strategies were modified for each database to account for their particularities. The search terms are detailed in Additional file 1: Appendix 1. For the selection of articles, first abstracts were screened and then full-texts. The detailed inclusion and exclusion criteria for the abstract and full-text screening are outlined in Table 2. Once the full-texts were identified, general descriptive data about each paper (such as authors, year, and journal) as well as relevant information related to the components and dimensions of the HIS Evaluation Framework were extracted. For the latter, the primary interest was on how authors reflected and discussed their findings in light of the system. For instance, the time point of data collection (baseline and follow-up) was frequently stated in the Methods section of the papers. Any critical reflection in the papers upon these time points was of interest for the present study. The first author conducted the data extraction, the second author reviewed and challenged the extracted data on a regular basis; the data extraction sheet was then presented to an expert in PROMs (measurement) and HIS to gain his feedback on the plausibility of the information extracted and enhance credibility. No quality check of the identified studies was conducted since the aim was not to synthesize the previous results qualitative or quantitatively but to extract supporting and hindering factors of the HIS.

**Table 2:** In- and exclusion criteria for systematic search and review

Inclusion criteria:
Swedish Hip or Knee Arthroplasty Registers
- reference to Swedish Hip or Knee Arthroplasty register or related data set
AND reference to integration or utilization of PROMs in the registries
AND
- primary research
- published in English language
- access to the full-text
NHS PROMs Programme
- reference to the NHS PROMs programme or related data set
AND
- primary research
- published in English language
- access to the full-text
Exclusion criteria:
- reference to other registries OR other Quality Outcomes Framework PROMs efforts
- other registries, such as the National Joint Registry
- secondary research, e.g. systematic literature review, books
- conceptual papers to inform or challenge PROMs’ development
- studies conducted to inform the development of the NHS PROMs programme e.g. published before the Swedish Arthroplasty Register or NHS PROMs programme existed

#### Part 2 expert interviews

In the context of this study people were considered as experts if they have been involved in research based on or related to the selected systems. To identify experts, research networks were identified using social network analysis [22]. In this study, a network reflects a group of people who have collaborated on work related to the system. The more a person has been involved in work related to the systems, the more central the person appears in the network. The networks were created based on the author information extracted from the literature identified in the first part of this study. The social network analysis for both countries respectively was conducted using the graph components of the networkx package of Python. Publications were iteratively analysed. For each individual publication all authors were inserted as nodes into the graph if they were not already existing in the graph. Each author pair (with n authors, one has n*(n-1)/2 pairs) represents an edge with weight 1 in the graph between the two corresponding authors. If an edge exists already, then the weight of this edge is incremented by 1. The networks, which resulted from this analysis, served as the foundation to identify experts with varying expertise.

From each identified network at least one person was invited for an interview. The intention was to interview 6 to 9 experts for each country. Twelve to 14 people were initially contacted. The guiding principles were: i) from each network authors with the highest number of publications would be contacted first; ii) the number of persons contacted from each network should be reflective of the actual network size. This approach allowed experts that are representative for the different research networks to be invited. Potential participants were contacted via e-mail with information about the study and an invitation to participate in a phone interview on a date of their preference. An information sheet was attached to the e-mail which outlined the purpose of this study, information about the data collection and analysis process, as well as data storage. Two reminders, the first after 4 weeks and the second after 8 weeks, were sent if no response was received.

The interview started with an open question about the goals of the specific system and whether the participant’s considered they had been achieved. Targeted questions were then asked based on the results of the literature review. This approach to developing an interview guide is consistent with qualitative research using directed content analysis [23] which was applied in this study (see Data analysis). A pilot interview was conducted with a person familiar with either of the two country examples. The interview guide was revised based on the feedback from the pilot interview. The final interview guide (see Additional file 2: Appendix 2 for the NHS PROMs Programme and Additional file 3: Appendix 3 for the SHPR and SKAR) was sent one day prior to the interview to each participant to remind him or her about the phone interview and provide some information about the main topics to be addressed. During the interview, the researcher elaborated on the background information relating to each aspect. For instance, response bias was identified as an aspect related to system quality and listed as a targeted question in the interview guide. During the interview, the researcher provided further information on the numbers and characteristics of non-responders, potential reasons for and threads due to this bias as described in the literature to stimulate the discussion. The interviews were tape-recorded after receiving verbal consent of participants at the beginning of the interview.

### Data analysis

#### Part 1 systematic search and review

A directed content analysis was conducted [23]. Such type of analysis is suitable when some research about a topic exists already and can serve as the foundation for further research. In this study, the Health Information System Evaluation Framework served as the foundation to identify themes to be subsequently presented to experts in the interviews using directed content analysis. The components and dimensions of the Health Information System Evaluation Framework deployed in this study served as the guidance for the initial coding. The analysis of the literature was performed in a first step for the two country examples separately, and in a second step across examples. The identified papers were reviewed and relevant paragraphs, in particular from the Discussion sections, were assigned to the components and dimensions of the framework. Subsequently, each component and dimension along with the assigned paragraphs, were reviewed and more general themes identified. The identified themes informed the targeted questions for the interview.

#### Part 2 expert interviews

The interviews were transcribed verbatim and then coded using directed content analysis [23] as described already for Part 1. Each transcript was read several times and relevant paragraphs assigned to the components and dimensions of the HIS Evaluation Framework [18]. For any information that could not be assigned to the framework a new theme was created. Each component and dimension, along with the assigned paragraphs, were then reviewed to identify more general themes. Subsequently, all themes were checked against each other to avoid redundancies across themes and ensure that the final themes are mutually exclusive. Once the themes were identified, they were reviewed and assigned to either being a supporting, hindering or neutral factor. This assignment was done initially by the first author, then reviewed by the second author and revised accordingly. As in the literature review, the analysis was conducted first for the two country examples separately and then across examples.

## Results

### Part 1 systematic search and review

**Fig. 2:**
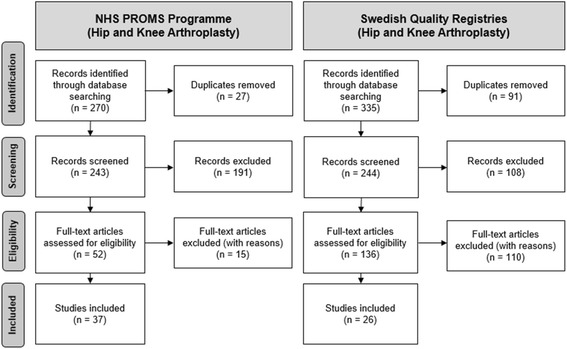
Results of literature search

The electronic search resulted in 270 identified records for the NHS PROMs Programme and 335 records for the SHPR and SKAR together. After removing duplicates and applying the in- and exclusion criteria, 37 records for the NHS PROMs Programme and 26 records for the SHPR and SKAR were included in the further analysis. The details of the literature search flow are presented in Fig. 2.

For the NHS PROMs Programme, papers were mainly excluded because they described studies which informed the development of the NHS PROMs Programme or other initiatives to collect PROMs data and enhance quality through PROMs but not grounded within the NHS PROMs Programme. For the SHPR and SKAR, the main reasons for excluding papers were reference to other registries, no reference to PROMs, reference to either registry in the discussion but the registry was not a fundamental component of the study itself.

The directed content analysis revealed aspects of each component of the HIS Evaluation Framework. The most salient themes included aspects related to governance and funding, uptake of information by different stakeholders, the selection of PROMs and the linkage of data with other databases. These themes were then presented to experts within the scope of the interviews for further discussion. Throughout the interview, detailed findings from the literature review related to each theme and for the respective country example were presented by the researcher.

### Part 2 expert interviews

**Fig. 3:**
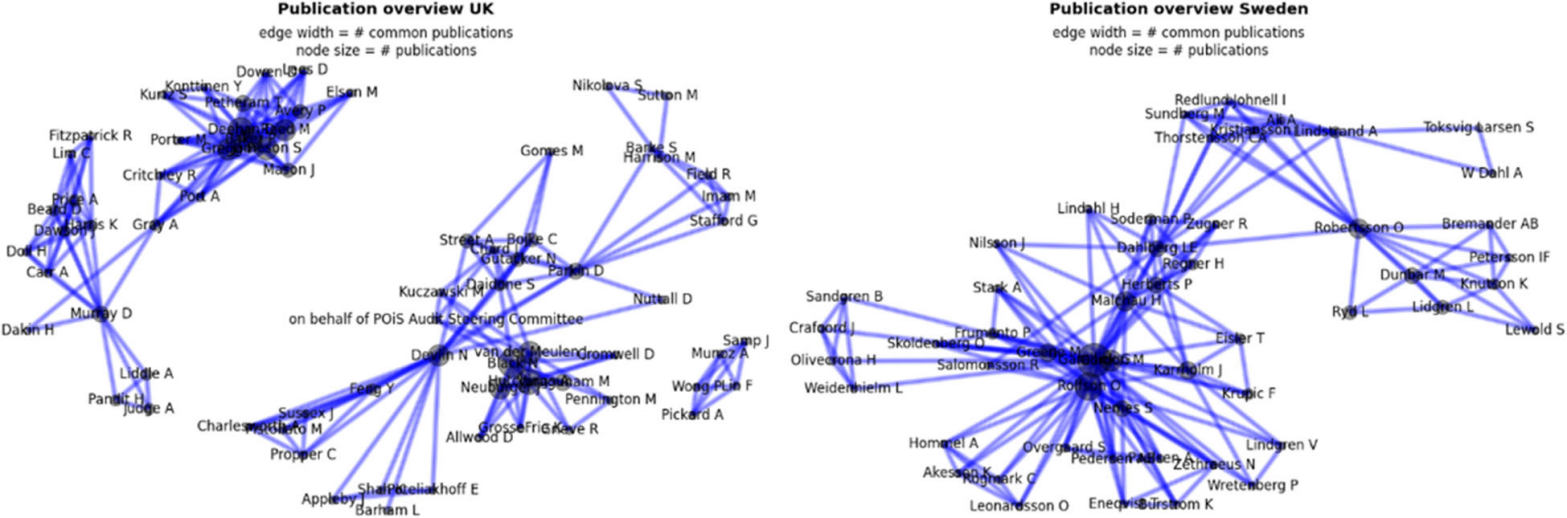
Results from Social Network Analysis

*Experts – NHS PROMs Programme.* The social network analysis for the NHS PROMs Programme resulted in 5 networks as illustrated in the left part of Fig. 3. The networks had their main foci on economics, clinical practice, epidemiology and psychometry. Two networks reflected two research teams of which each published one paper. Out of the 13 people invited, 7 participated, 6 did not respond or declined. Experts included clinicians, economists, epidemiologists, and statisticians.

*Experts – SHPR and SKAR.* The social network analysis for the SHPR and SKAR resulted in one network. The analysis revealed research collaborations across the two registries as represented in the right part of Fig. 3. Thus, in this case experts were invited not only based on the highest number of publications, but so that they were representative for the hip and knee registries. Out of 13 people who were invited for an interview, 2 attended a phone interview, one from SHPR and one from SKAR, and 1 expert from the SKAR responded via e-mail. Six did not respond and 4 declined or referred to others who already agreed to participate. All experts who participated in the interviews were clinicians by background; only one worked primarily as such at the time of the interview.

All interviews for both, the UK and SE system were conducted between end of February and April 2016 and lasted on average 51 min (min. 25 min – max. 1,5 h).

*Interviews.* The majority of the experts highlighted that the integration of the PROMs into the HIS and its success has to be considered in light of the goal for which the data collection was set up. The experts for the SHPR and SKAR highlighted that the goals of the registry, namely to improve quality of care, did not change through the integration of the PROMs. One expert of the SKAR highlighted that the integration of the PROMs allowed the registry to realize a bio-psych-social perspective in evaluating and monitoring outcomes which is important since

*“We are operating people, not knees.” (SE_3_L111).*

The experts with reference to the NHS PROMs Programme emphasized that the goal of the programme was broadly and vaguely defined whereby improving quality of care was one goal amongst others. Most experts highlighted that the program demonstrated that a nation-wide PROMs data collection is feasible but the achievement of other goals, such as improving quality of care, remain yet to be proven.

The factors related to each system which revealed from the analysis are detailed by components of the HIS Evaluation Framework in Tables 2, 3, 4, 5 and 6. For the two Swedish registries, it is explicitly stated if a statement was only provided for one registry. No inferences can be made that this does or does not apply to the other registry since no information on the topic was given by the experts of the other registry. Points that were controversially discussed in relation to each component are elaborated on in the subsequent sections. Only results are presented that revealed of relevance in the analysis across the country examples.

**Table 3:** Results of expert interviews related to the component Organization of the HIS Evaluation Framework

NHS PROMs Programme	Swedish Quality Registries: SHPR and SKAR
*Governance*	
+ Investment and commitment of government, including funding to get programme started + Clear ownership of programme by NHS England + Standardized data collection protocols	+ SHPR: partly government funded which makes it possible to employ people to sustain, improve, and further develop the registry
- Reforms within the NHS put responsibility for programme at question for some time which, in turn, weakened the programme - Lack of ownership by clinicians and risk that data is ignored by them given their limited involvement in setting up and running the programme	- SHPR: government funding does not cover research activities
	○ Limitations of PROMs data in the context of registries need to be taken serious since the data provides foundation for health policy changes ○ Data governance: question whether data is individual or societal good needs to be clarified ➔ transparency on individual’s right to privacy versus the society’s mandate to provide high quality health care
*Capacity building*	
	+ Resources, incl. Government funding, are in place to build up the capacity to collect, analyse, disseminate and implement findings
- Stakeholders, in particular economists and staffing of hospital boards lack training in quality measurement and management - Dominance of financial matters over quality in hospitals - Skills and knowledge of an expert in quality management does not fit into a defined role within the NHS	

Supporting factors are indicated with a “+”, hindering factors with a “-”, and neutral statements related to the system, including considerations for the future with a “○”. For the two Swedish registries, it is explicitly stated if a statement was only provided for one registry. No inferences can be made that this does or does not apply to the other registry since no information on the topic was given by the experts of the other registry

**Table 4:** Results of expert interviews related to the component Human of the HIS Evaluation Framework

NHS PROMs Programme	Swedish Quality Registries: SHPR and SKAR
*Reporting*	
	+ Established infrastructure for developing and disseminating annual reports + Annual registry meeting with key stakeholders i) to gain feedback on and finalize annual report, and ii) to discuss potential future directions of the registry, beneficial further analyses etc. + Clinicians provide feedback at the annual registry meeting on whether they understand what is presented in the reports accurately and how they would interpret the figures
- Central efforts to provide data at the level of individual hospitals, surgical teams and surgeons are lacking - Led by economists with the aim to save costs rather than to improve quality	
- Reports allow to understand where hospital stands relative to anyone else but not to identify deficiencies in care - Lack of resources, incl. Funding, for analysing, disseminating (e.g. user-friendly outputs) and implementing data (e.g. support local staff to understand reports) – most resources went into data collection ○ Most reports provide data at level of commissioning groups or NHS trusts	
*Stakeholder engagement*	
	+ Main forum to engage with stakeholders is annual registry meeting which is attended by one clinician representative of each hospital
○ Need for tailoring information to and training for respective stakeholder group to ensure best possible uptake ○ PROMs data has potential to respond to questions that matter to patients such as What will the outcome be for me? or What are my changes to get better? ○ Limited investment to promote information to General Practitioners who would be well suited to use PROMs data for shared decision making and referral behaviour ○ Need to get surgeons engaged to examine their practices and outcomes ○ Programme provides database for research, however, bureaucracy becomes increasingly difficult and time consuming for accessing the data ○ Other stakeholders mentioned include economists, commissioners, researchers, NHS England, etc.	

Supporting factors are indicated with a “+”, hindering factors with a “-”, and neutral statements related to the system, including considerations for the future with a “○”. For the two Swedish registries, it is explicitly stated if a statement was only provided for one registry. No inferences can be made that this does or does not apply to the other registry since no information on the topic was given by the experts of the other registry

**Table 5:** Results of expert interviews related to the component Technology of the HIS Evaluation Framework

NHS PROMs Programme	Swedish Quality Registries: SHPR and SKAR
*Selection of PROMs*	
○ Generic and health condition specific measure complement each other ○ Generic measure in general, and EQ-5D in particular + enables comparisons across health conditions, procedures, and countries + can be transformed into a utility measure which is most valuable for economic analyses - won’t pick up much variation - won’t pick up the impact of a specific health condition on a person’s overall life and well-being	
*Data collection of PROMs*	
+ Data linkage with the National Joint Registry is important – adds value with the 1, 3 and 5 years follow-up	
○ Time points for data collection need to be standardized e.g. Baseline: it matters whether the questionnaire is filled in when the decision of surgery is made or when the patients shows up for surgery. In some countries the time in between these time points may be more than a year; how much patients deteriorated in this time would be an important question in itself but challenges comparability if the time point is not standardized.	
○ Follow-up 6 months post-surgery Though one expert stated it is effectively 6–10 months post-surgery - From clinical point of view: too short + From a quality management point of view, reasonable time frame to collect, analyse the data and return the results to the hospitals so that they can act upon them	○ Follow-up for 1 and 6 years post-surgery Rationale for 1 year: to ensure patient is rehabilitated properly Rationale for 6 years: time when most of the complications, such as loosening, may start to occur ○ SHPR: Follow-up mechanism created coincidently a channel for communication between patients and clinicians: when patients return the follow-up questionnaires to the clinic they comment on how they are doing and provide occasionally feedback on the procedures
*Data linkage*	
	+ Unique Swedish Identifier Number is of great value for the linking with other Swedish databases, e.g. inpatient registry, prescribing drug registry, or spine registry + Linkage with databases in other countries is particularly valuable for research questions related to rare diagnoses or prostheses
- Linkage is not always straightforward since there is no unique identifier	
- Logistics, bureaucracy and ownership of the data complicates linkage ➔ expert suggested that ideally all national data collection efforts would be under guidance and jurisdiction of one organization	
○ NHS PROMs Programme was set up to not duplicate but rather complement already existing databases, e.g. Hospital Episode Statistic, National Joint Registry ➔ linkages with these datasets are essential to generate a comprehensive database with various socio-demographic information and information related to the health condition and intervention ○ Some experts suggested to integrate the PROMs data collection into routine hospital records rather than having a separate database	

Supporting factors are indicated with a “+”, hindering factors with a “-”, and neutral statements related to the system, including considerations for the future with a “○”. For the two Swedish registries, it is explicitly stated if a statement was only provided for one registry. No inferences can be made that this does or does not apply to the other registry since no information on the topic was given by the experts of the other registry

**Table 6:** Results of expert interviews related to the component Net benefits of the HIS Evaluation Framework

NHS PROMs Programme	Swedish Quality Registries: SHPR and SKAR
*System-specific considerations*	
+ Health systems overall become more reliant on health information which encourages the collection of high quality information such as in the NHS PROMs Programme + The programme highlights a positive shift in health care toward integrating patient-reported information and objective measures + Benefit case studies are produced to stimulate the implementation of the results from the programme into practice	
○ Efforts are needed in the future to inform various stakeholder groups about the availability of the data and how it could be used to inform their practices. ○ For future analyses, examining not only variations between hospitals but also between clinical teams was recommended ○ The next phase of the NHS PROMs Programme should be an extension to long-term conditions, mental health and emergency admissions. Long-term conditions and mental health are challenging since they involve various interventions potentially across settings. Emergency care is challenging since one needs to find consensus on what are you actually measuring: improve state or restore state the state in which a patient was in a week earlier.	○ Collecting information on other constructs, such as catastrophizing, is something to be considered potentially for the future. Catastrophizing is one example of a construct which is a fixed personality trait which can be highly influential on the outcome, yet does not change due to the surgery ○ Adding new concepts and themes is not appropriate for large-scale application since it implies more burden on respondents, but should be considered on the level of research ○ Registries serve as the foundation for developing guidelines e.g. on reducing infections ○ Data is used by responsible people for resource allocation to examine their resource use in comparison to other counties and act upon these findings

Supporting factors are indicated with a “+”, hindering factors with a “-”, and neutral statements related to the system, including considerations for the future with a “○”. For the two Swedish registries, it is explicitly stated if a statement was only provided for one registry. No inferences can be made that this does or does not apply to the other registry since no information on the topic was given by the experts of the other registry

#### Organization

The SHPR and SKAR built upon decades of experience in building up the infrastructure for the registries, and thus reported about a rather consolidated governance and infrastructure as reflected in Table 3, left column. In contrast, the experts related to the NHS PROMs Programme reflected more critically upon these themes since they considered the system to be in its infancy and the governance as decisive for the success and sustainability of the programme (Table 3, right column). For instance, experts suggested that more involvement from clinicians would have been beneficial from the very beginning on. However, some argued that it would have required more time resources to agree upon the data collection protocols back then since there was a lack of outcome measurement conducted in routine practice. Overall, a lack of capacity for quality management within the NHS was identified.

*“It is the responsibility of the health care system in general to develop staff so that they can manage quality and we don’t have that. (…) There isn’t senior leadership in hospitals with responsibility and the skills and knowledge as to how to improve quality. So in a way what we got is increasingly sophisticated national data getting produced. But actually it is falling on stony ground because people don’t know because they haven’t been trained how to use it.” (UK_1_L207–213).*

#### Human

The reporting and engagement with stakeholders revealed as important themes. Both are measures of the success of an information system according to the HIS Evaluation Framework. The detailed findings related to these themes are presented in Table 4. The standard way of reporting results of the NHS PROMs Programme is with funnel plots. Prior to preparing the plots, the data is adjusted for case-mix. Different opinions amongst experts revealed on whether the data is easy to analyse and interpret:

*“There is enough technical guidance for someone reasonable capable to deal with PROMs data – it is pretty straight forward.” (UK_2_L252–253).*

*“I think the problem with this – as I see it – if the aim is to provide more data for patients and commissioners to increase choice and accountability, I think the reporting processes presently aren’t robust enough to allow that to happen. By that I mean the data that comes out is difficult for commissioners and patients to interpret, it is statistical analysis of it, and the requirements of the statistical analysis to ensure that the data is analysed is appropriately are probably too complex for commissioners and patients to understand.” (UK_4_L11–16).*

This complexity and related lack of transparency impedes end-users to engage with the data:

*“You can’t dig into it, you can’t ask questions of it. You have just take on face value of what they give you, which is a bit disappointing. Unless you can dig into it and understand the data, you cannot identify the area in which you need to improve to get better.” (UK_4_L325–327).*

#### Technology

All participants, irrespective of the system, stressed that as a basic principle the questionnaires should be not too long to keep the burden on participants in an acceptable range:

*“Simple data collected comprehensively is better than comprehensive data collected simply or poorly.” (SE_3_L140–141).*

Experts from both country examples mentioned some alternative PROMs but in consideration of their length and popularity considered the current selection as appropriate. Next to the aspects which are shown in Table 5, the majority of experts challenged whether it is appropriate to apply existing population preferences to national PROMs data. In particular if such data is then used to inform the treatment of individual patients. For instance, for the EQ-5D existing population preferences are used to transform the scores into utility measure. Some experts argued that preferences specifically tailored to the purpose of the NHS PROMs Programme are needed. Such research efforts are ongoing. Others argued that the population preferences are informative for the individual patient, e.g. to know what the population feels about the trade-off between different dimensions covered by the EQ-5D, but should not be the basis for the final clinical decision making.

The NHS PROMs Programme includes a section where patients are asked about any complications in the context of the surgery. Experts were very critical upon these questions being patient-reported. Since differences in the interpretation of clinicians and patients in their judgment of complications are to be expected. While clinicians may introduce a systematic bias, e.g. by rating certain things always or never as complication, patients may overestimate complications. One expert stated that cross-linking the PROMs data with the Hospital Episode Statistics (HES) did not reveal much agreement. The HES contains data on complications as judged by clinicians.

The linking of PROMs data to other databases provided more of an added value to the SHPR and SKAR whereas it was rather essential for the NHS PROMs Programme to generate a reasonable data set. The aspects which revealed regarding linkage are listed in the bottom part of Table 5. Experts of the NHS PROMs Programme mentioned that some information is collected in various databases but with different modes. Examining how the data on one specific variable varies by administration mode is meaningful for the verification of the credibility of the data. Thus, data linkage does not only allow to extend the data but also to enhance its quality.

#### Net benefits

In addition to system-specific considerations listed in Table 6, one of the areas mentioned by all experts with controversial opinions is the benefit of PROMs for clinical management and decision making. To strengthen the usefulness of PROMs further for clinicians and patients, the SHPR is currently developing a decision support tool which will provide individualized information on a given patient’s risks and potential benefits of a surgery.

*“The information will be based on what we have in the registry. It is like patients like you generally have a 60 % chance of improving in usual activities in the EQ-5D. Given your responses now, and given your age, gender, socio-economic background, you would have this and this chances of improvement or risk of suffering from complication.” (SE_2_L169-L170).*

The Decision Support Tool will be available on the clinician’s portal; whether a patient portal would be created was still an open question at the time of the interview. Several experts of the NHS PROMs Programme mentioned that the integration of PROMs data into routine clinical practice for decision making would make another success of the programme. Nevertheless, other experts from both country examples challenged whether the PROMs used in these systems are appropriate for use in individual decision making since they were all developed for clinical trials to compare intervention groups against control groups. One expert from the NHS PROMs Programme stated that the data available now could serve to establish the baseline population and to develop trajectories of these patients to inform future practice.

*If there are different kinds of patients, you know, you can stratify it by gender or age, and by clinician type and communicate that kind of information is very meaningful to patients who fit that profile. I think this is part of evidence-based medicine. (UK_6_L77–80).*

The lack of such a baseline made another expert from the SKAR sceptical about the readiness of PROMs for use in clinical practice. The value of PROMs in managing access to care was also mentioned by experts from all systems. Most experts were sceptical since no evidence is available yet on the impact of such practices. Concerns were voiced that patients and clinicians may start gaming the system.

*“Patients would find out that we are stratifying on a filter questionnaire they will all game the questionnaire.” (SE_3_L467–468).*

*“We don’t know the impact how it is going to change the way how people are managed, how it is going to change (…*) *For instance surgeons might just want to choose patients who are going to do well definitely so that it looks good at them, so that they have good outcomes if it is something they are going to be judged on. And it can also go to the hospital level where you can have patients who actually need help but will not necessarily benefit based on the OKS. They might be left out of the system to their own devices which may bring extra cost to the overall health care etc.” (UK_5_L175–191).*

An important issue mentioned for the SHPR and the NHS PROMs Programme is the need to have immediate access to data once it is used for clinical decision making.

*“The issue is how quickly the data can be made available for surgeons, and also for clinicians and hospitals, commissioners group and those from practice side who would like to have high quality-data on patients as soon as possible.” (UK_2_L96–98).*

The cleaning and validating of the data does not take place real-time in either system and is essential for sustaining high data quality in the registry. The expert from the SHPR stressed that using the data for clinical decision making should not defeat the quality of the registry and its purpose, which is quality improvement and monitoring of outcomes.

## Discussion

This study examined supporting and hindering factors of the NHS PROMs Programme and Swedish Hip and Knee Arthroplasty Registry with respect to the integration of PROMs within the entire health system. The country examples are representative for a system that integrates PROMs into an existing HIS (SHPR and SKAR), and a system that has been set up independently and specifically for collecting PROMs data (NHS PROMs Programme). Based on the experts’ opinions, these country examples were successful in their efforts to collect PROMs within the entire health system.

PROMs assist in improving the quality of care according to experts of this study. The findings highlight the role of PROMs to realize a bio-psycho-social perspective on health and may constitute one important step toward going beyond solely biomedical outcomes in improving quality of care. This is in line with the argument previously made that PROMs have the potential to stimulate the move beyond traditional outcomes of mortality and morbidity [7]. Nevertheless, it is also fair to say that based on the findings of this study the value of PROMs for improving quality of care remains yet to be established in the UK. One of the main concerns for using PROMs in monitoring quality of care is the lack of an established standard on what change in PROMs scores should be achieved when conducting a surgery for arthroplasty. Such knowledge is needed if PROMs are to be used for monitoring the quality of care since “quality is not represented by health status but by the extent to which improvement in health status that are possible are realized” ([24]; p 10). The existing data sets collected within these country examples could serve to establish evidence on what change can be expected.

Though all experts highlighted the value of using the PROMs data collected within these systems in routine clinical practice, this study sheds light on some important considerations. The combination of generic and health-condition specific instruments suggested previously [13] is also supported by the experts who participated in this study. This combination provides clinical face validity for treating patients with a particular health condition and enables comparisons across health conditions. However, experts challenged whether the data collected within the country examples is reliable for routine clinical decision-making, since the reliability of these instruments has been tested previously mainly on group level. Concerns were also mentioned with regard to the use of PROMs to guide access to care. Furthermore, as already highlighted by Boyce, Browne and Greenhalgh [9], the infrastructure for collecting and retrieving PROMs data revealed also in this study as a main source for success or failure for integrating PROMs into routine practice. The need for finding a balance between providing high data quality and making the data available instantly for routine use was identified as challenging. Future research may address some of these open questions on the use of the PROMs data collected within these systems in routine clinical practice.

The main difference between the country examples refers to the organization of the systems, in particular their governance. The NHS PROMs programme enacts a top-down approach whereas the Swedish registries a bottom-up approach. Various stakeholders, including commissioners, economists, patients, and surgeons were identified for the NHS PROMs programme. The variety of the stakeholders of the NHS PROMs Programme could be seen as strength, though experts in this study identified the need for providing reports that are better tailored to the needs of the different stakeholders. Having commitment of the government, in particular with respect to funding, was identified as supporting factor of the NHS PROMs Programme and a weakness of the Swedish registries. The top-down approach deployed in the NHS PROMs Programme resulted in a lack of ownership of the data by clinicians. In contrast, clinicians were the key stakeholders stated for the Swedish country examples. They were involved in finalizing reports. The limited involvement of clinicians in the NHS PROMs Programme was identified as a thread for its sustainability. Taking the identified supporting and hindering factors of both country examples into account, a participatory approach with involvement and commitment of stakeholders at the clinical and managerial level would be ideal. Such comprehensive approach would be also in line with knowledge on the successful implementation of innovative programs in health care [25].

The credibility of the findings of this study need to be considered in light of the methods deployed. Reviewing the literature systematically to inform the interview guide can be considered as a method triangulation to enhance the rigor of this study [26]. All experts stated that they consider the most relevant themes captured by the interview guide which was taken as confirmation of the comprehensiveness of the review. The selection of experts based on the social network analysis allowed identifying experts with various areas of expertise (see Table 4). Nevertheless, the basis for generating the networks was a pool of people who have published internationally any work in relation to any of the two systems. Thus, for future research it is recommended to include various stakeholders, including politicians, patients and service users of these systems. Noteworthy, this study only focused on two selected information systems with a focus on two specific clinical populations from two different countries. Similar systems do exist for other health conditions and in other countries. Thus, also additional information systems and their relevant stakeholders need to be included in future research.

The unequal number of experts related to the NHS PROMs Programme and the Swedish registries is reflective of the organizational structure of these systems. The SKAR and SHPR are organized around a register centre which is responsible for the continuous registry work, including clinical research. Expert affiliated with the register centre of the SKAR and SHPR participated in this study. Given their position in relation to the registers, it can be assumed that they have a comprehensive knowledge about the supporting and hindering factors of these registers.

Recommendations to overcome some of the weaknesses of phone interviews in comparison to face-to-face interviews were integrated into this study [27]. For instance, participants received a reminder of the time of the interview a day prior to the interview along with an overview of the interview guide. This approach allowed re-confirming the date and timing and getting the participants into the scope of the interview. Furthermore, the speakerphone/tape recording method, which was used in this study, was tested prior to the interviews [28].

Directed content analysis [23] foresees the use of theory as guidance in the initial coding. It has been argued that this method may introduce a strong bias into the results since it is easier to find evidence that is supportive rather than unsupportive of the theory. In this study, the HIS Evaluation Framework [18] served as the frame of reference to ensure that a comprehensive perspective on HISs is enacted and all components relevant for a HIS are taken into consideration. No theoretical guidance on how these components and dimensions are intended to interact were adopted from previous research in this study.

## Conclusions

Both country examples succeeded in integrating PROMs into HIS on a national scale. The value of adding the patient perspective in quality improvement by integrating PROMs in HIS tailored toward this purpose was stressed by experts from both country examples. Though the systems examined in this study are different in their historical development and the political context in which they are embedded in, there was agreement amongst experts that any such system is valuable and needs to be kept simple and clear. The lack of an established standard on what change PROMs should be achieved by an intervention was considered as a limitation of the usefulness of the PROMs for monitoring quality of care. Whether the PROMs data collected within these systems can be used in routine clinical practice is considered a challenge in both countries. The findings of this study can inform the further development of the existing systems as well as the implementation of such systems in other health conditions or settings. For the development of similar HISs in the future, a participatory approach with involvement and commitment of stakeholders at the clinical and managerial level can be recommended based on the findings of this study.

## Additional files

Additional file 1: Appendix 1. Search terms for systematic literature reviw. Provides an overview of the search terms used for the systematic literature review for each database and country separately. (DOCX 13 kb)

Additional file 2: Appendix 2 Interview guide NHS PROMs Programme. Shows the interview guide used for the interviews with experts related to the NHS PROMs Programme. (DOCX 14 kb)

Additional file 3: Appendix 3 Interview guide Swedish Hip or Knee Arthroplasty Registries. Shows the interview guide used for the interviews with experts related to the Swedish Hip or Knee Arthroplasty Registries. (DOCX 14 kb)

## Abbreviations

EQ-5D: Health-related quality of life measure developed by the EuroQoL Group
HES: Hospital Episode Statistics
HIS: Health Information System
ISO: International Organisation for Standardization
KOOS: Knee injury and Osteoarthritis Outcome Score
NHS: National Health Service
OHS: Oxford Hip Score
OKS: Oxford Knee Score
PROMs: Patient-reported outcome measures
SHPR: Swedish Hip Arthroplasty Register
SKAR: Swedish Knee Arthroplasty Register
