# Supplementary material for: Improving quality of care through patient-reported outcome measures (PROMs): expert interviews using the NHS PROMs Programme and the Swedish quality registers for knee and hip arthroplasty as examples
Source: BMC Health Serv Res. 2018 Feb 7;18:87. doi: 10.1186/s12913-018-2898-z (PMC5803859; doi:10.1186/s12913-018-2898-z)
Supplement: Supplementary file 1 — Appendix 1. Search terms for systematic literature reviw. Provides an overview of the search terms used for the systematic literature review for each database and country separately. (DOCX 13 kb) [file 12913_2018_2898_MOESM1_ESM.docx]

| Sweden | |
| --- | --- |
| PubMed | (Swedish[All Fields] OR ("sweden"[MeSH Terms] OR "sweden"[All Fields])) AND (("registries"[MeSH Terms] OR "registries"[All Fields] OR "registry"[All Fields]) OR ("Regist Ky Hist Soc"[Journal] OR "register"[All Fields])) AND ("Hip Arthroplasty"[All Fields] OR "Knee Arthroplasty"[All Fields]) |
| SCOPUS | TITLE-ABS-KEY ( ( swedish  OR  sweden )  AND  ( registry  OR  register )  AND  ( hip  arthroplasty  OR  knee  arthroplasty ) ) |
| UK | |
| PubMed | (NHS[All Fields] AND ("federal government"[MeSH Terms] OR ("federal"[All Fields] AND "government"[All Fields]) OR "federal government"[All Fields] OR "national"[All Fields]) AND PROMs[All Fields] AND Programme[All Fields]) OR ((NHS[All Fields] OR UK[All Fields] OR ("great britain"[MeSH Terms] OR ("great"[All Fields] AND "britain"[All Fields]) OR "great britain"[All Fields])) AND (("patients"[MeSH Terms] OR "patients"[All Fields] OR "patient"[All Fields]) AND ("research report"[MeSH Terms] OR ("research"[All Fields] AND "report"[All Fields]) OR "research report"[All Fields] OR "reported"[All Fields]) AND ("outcome assessment (health care)"[MeSH Terms] OR ("outcome"[All Fields] AND "assessment"[All Fields] AND "(health"[All Fields] AND "care)"[All Fields]) OR "outcome assessment (health care)"[All Fields] OR ("outcome"[All Fields] AND "measure"[All Fields]) OR "outcome measure"[All Fields])) AND (("arthroplasty, replacement, hip"[MeSH Terms] OR ("arthroplasty"[All Fields] AND "replacement"[All Fields] AND "hip"[All Fields]) OR "hip replacement arthroplasty"[All Fields] OR ("hip"[All Fields] AND "replacement"[All Fields]) OR "hip replacement"[All Fields]) OR ("arthroplasty, replacement, knee"[MeSH Terms] OR ("arthroplasty"[All Fields] AND "replacement"[All Fields] AND "knee"[All Fields]) OR "knee replacement arthroplasty"[All Fields] OR ("knee"[All Fields] AND "replacement"[All Fields]) OR "knee replacement"[All Fields]))) AND ("1999/01/01"[PDAT] : "2015/12/31"[PDAT]) |
| SCOPUS | TITLE-ABS-KEY ( ( nhs  OR  uk  OR  great  britain )  AND  ( patient  reported  outcome  measures )  AND  ( "hip replacement"  OR  "knee replacement" ) ) |
