# Supplementary material for: Improving quality of care through patient-reported outcome measures (PROMs): expert interviews using the NHS PROMs Programme and the Swedish quality registers for knee and hip arthroplasty as examples
Source: BMC Health Serv Res. 2018 Feb 7;18:87. doi: 10.1186/s12913-018-2898-z (PMC5803859; doi:10.1186/s12913-018-2898-z)
Supplement: Supplementary file 2 — Appendix 2 Interview guide NHS PROMs Programme. Shows the interview guide used for the interviews with experts related to the NHS PROMs Programme. (DOCX 14 kb) [file 12913_2018_2898_MOESM2_ESM.docx]

**Improving quality of care through patient-reported outcome measures (PROMs)**

You have been invited to this interview since you have conducted research related to the *NHS PROMs Programme*. This study aims to examine how patient-reported outcome measures (PROMs) are integrated and utilized in existing health information systems (HIS) tailored toward improving quality of care using the NHS PROMs Programme with specific focus on knee and hip replacement, and the Swedish Knee and Hip Arthroplasty Registries as two examples. The details of the project are outlined in the adjunct project overview.

Kindly note, that this interview will be audio-recorded and subsequently transcribed verbatim. Data will be anonymized immediately after the interviews and deleted once this study has been completed. As experts have been selected purposefully, basic socio-demographic information about your professional position, geographical location and role related to the HIS may allow other experts to disclose your identity. Thus, upon completion of data collection, you will receive the socio-demographic information to be included about your person in any publication related to this study for your approval. *Do you have any further questions about the study and its process?*

The following points have been identified based on published literature on the NHS PROMs Programme and I will provide further context to these points throughout the interview. *You are not expected to respond to all of these aspects.*

**General Opening Question**

The first question is a general question regarding the NHS PROMs Programme. The programme has been set out to achieve several goals, including the evaluation and improvement of quality of care, estimating the amount of health gain for populations, and facilitating patient choice and provider accountability. *From your point of view, has the NHS PROMs Programme achieved its aims? Where do you see the main value of the programme?*

- **Technological characteristics**

Aspects related to service and system’s quality include:

- - Linkage of NHS PROMs Programme database with other databases, e.g. Hospital Episodic Statistics
  - Response bias
  - Exclusion of providers with only few cases

Aspects related to information quality:

- - Selection of PROMs
  - Timing of data collection
  - Self-report of complication rates
- **Human characteristics**
  - Relevant aspects related to system use and user satisfaction include
  - Uptake of information by different stakeholders
  - Full-scale nation-wide PROMs Programme vs. random samples
- **Organizational characteristics** incl. governance structure
  - Top-down approach in setting up and running the programme
  - Ownership of data

**General Concluding Question:** *Are there any aspects not addressed so far?*

**Thank you for your time and support of this study.**
